# A diradical based on odd-electron σ-bonds

**DOI:** 10.1038/s41467-020-17303-4

**Published:** 2020-07-10

**Authors:** Wenbang Yang, Li Zhang, Dengmengfei Xiao, Rui Feng, Wenqing Wang, Sudip Pan, Yue Zhao, Lili Zhao, Gernot Frenking, Xinping Wang

**Affiliations:** 1 State Key Laboratory of Coordination Chemistry, Jiangsu Key Laboratory of Advanced Organic Materials, School of Chemistry and Chemical Engineering, Collaborative Innovation Center of Advanced Microstructures, Nanjing University, Nanjing, 210023 China; 2 Center of Materials Science and Engineering, Guangxi University of Science and Technology, Liuzhou, 545006 China; 3 Institute of Advanced Synthesis, School of Chemistry and Molecular Engineering, Jiangsu National Synergetic Innovation Center for Advanced Materials, Nanjing Tech University, Nanjing, 211816 China; 4 Fachbereich Chemie, Philipps-Universität Marburg, Marburg, D-35032 Germany

**Keywords:** Chemical bonding

## Abstract

The concept of odd-electron σ–bond was first proposed by Linus Pauling. Species containing such a bond have been recognized as important intermediates encountered in many fields. A number of radicals with a one-electron or three-electron σ-bond have been isolated, however, no example of a diradical based odd-electron σ-bonds has been reported. So far all stable diradicals are based on two *s*/*p*-localized or *π*-delocalized unpaired electrons (radicals). Here, we report a dication diradical that is based on two Se∴Se three-electron σ–bonds. In contrast, the dication of sulfur analogue does not display diradical character but exhibits a closed-shell singlet.

## Abstract (web summary)

Stable diradicals are generally based on two *s*/*p*-localized or π-delocalized unpaired electrons (radicals). Here, the authors report a dication diradical that is based on two Se∴Se three-electron σ-bonds.

## Introduction

**Fig. 1:**
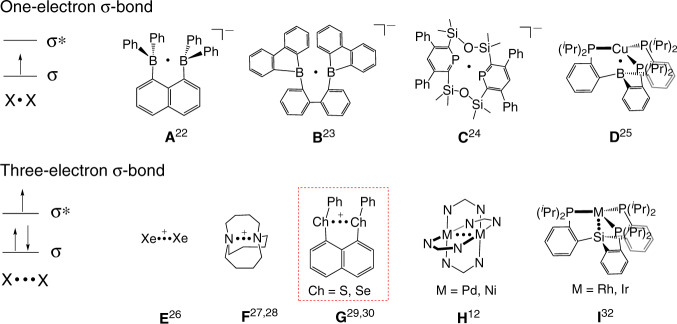
Schematic representation of odd-electron σ-bonds and selected examples. **a** The B·B one-electron σ–bond proved by EPR. **b** The B·B one-electron σ–bond proved by X-ray diffraction. **c** The P·P one-electron σ–bond. **d** The Cu·B heteronuclear one-electron σ–bond. **e** The Xe·Xe one-electron σ–bond. **f** The N∴N three-electron σ–bond. **g** The S∴S and Se∴Se three-electron σ–bonds. **h** The Pd∴Pd and Ni∴Ni three-electron σ–bonds. **i** The Rh∴Si and Ir∴Si heteronuclear three-electron σ–bonds.

Radicals are species that possess an unpaired electron^1–7^. In general, there are two classes of stable radicals: *s*/*p*-localized and *π*-delocalized. In the former, the unpaired electron resides on an *s*/*p*-orbital of one atom, while in the latter, the unpaired electron is *π*-delocalized over two or more atoms. Apart from these two classes, there is the third class of radicals that have an unpaired electron delocalized in an σ orbital or antibonding σ* orbital between two atoms, leading to a one-electron σ-bond and a three-electron σ-bond (Fig. 1), respectively. The concept of the odd-electron σ-bond was first proposed by Pauling^8^, and species with these intriguing bonds have been recognized as important intermediates in chemistry and biochemistry^9–21^. A number of radicals with a one-electron^22–25^ or three-electron σ-bond^12,26–32^ have been isolated and structurally studied (Fig. 1).

Diradicals are species with two unpaired electrons (radicals), which are of importance both in understanding of bonding nature and application as functional materials^33–37^. So far all stable diradicals are based on two *s*/*p*-localized or *π*-delocalized unpaired electrons (radicals); however, no example of a diradical based odd-electron σ-bonds has been reported. In 2014, we isolated selenium and sulfur radical cations (NapSe_2_Ph_2_)^+^ and (NapS_2_Ph_2_)^+^ (highlighted in Fig. 1)^29,30^ that feature a Se∴Se and S∴S three-electron σ-bond, respectively.

**Fig. 2:**
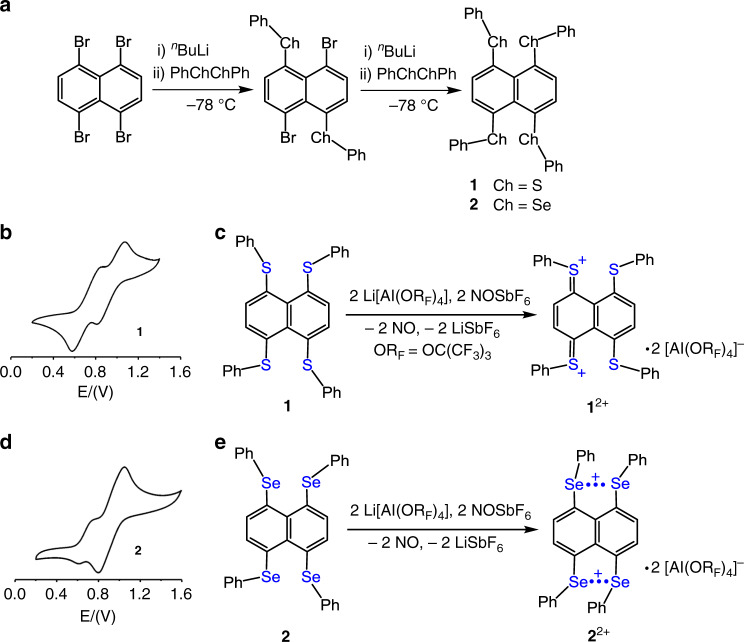
Preparation, cyclic voltammograms, and two-electron oxidation of tetrachalcogenides **1** and **2**. **a** Preperation of compounds **1** and **2**. **b** The cyclic voltammetry of **1**. **c** Two-electron oxidation of **1**. **d** The cyclic voltammetry of **2**. **e** Two-electron oxidation of **2**.

We now report a diradical (**2**^2+^, Fig. 2) that is based on two Se∴Se three-electron σ-bonds. In contrast, the dication of sulfur analog (**1**^2+^) does not display diradical character but exists as a closed-shell singlet instead.

## Results

### Syntheses of dications

Tetrachalcogenides **1** and **2** were synthesized in two steps from 1,4,5,8-tetrabromo naphthalene^38^ and ^*n*^BuLi with corresponding diphenyl dichalcogenide at −78 °C, respectively (Fig. 2). Their cyclic voltammetry (CV) in CH_2_Cl_2_ at room temperature with supporting electrolyte ^*n*^Bu_4_NPF_6_ displays two reversible oxidation peaks at oxidation potentials of +0.84, 1.07 V (**1**) and +0.74, 1.04 V (**2**) (Fig. 2). Prompted by CV data, **1** and **2** were treated with two equivalents of Li[Al(OR_F_)_4_] (OR_F_ = OC(CF_3_)_3_)^39^ and NOSbF_6_ in CH_2_Cl_2_ to afford dications **1**^**2+**^ and **2**^2+^ in modest yields, respectively (Fig. 2). These dications are air sensitive but thermally stable under nitrogen or argon atmosphere. They were characterized by chemical analysis, UV absorption spectroscopy, EPR spectroscopy, single-crystal X-ray diffraction, and superconducting quantum interference device (SQUID) measurements.

### Crystal structures

**Fig. 3:**
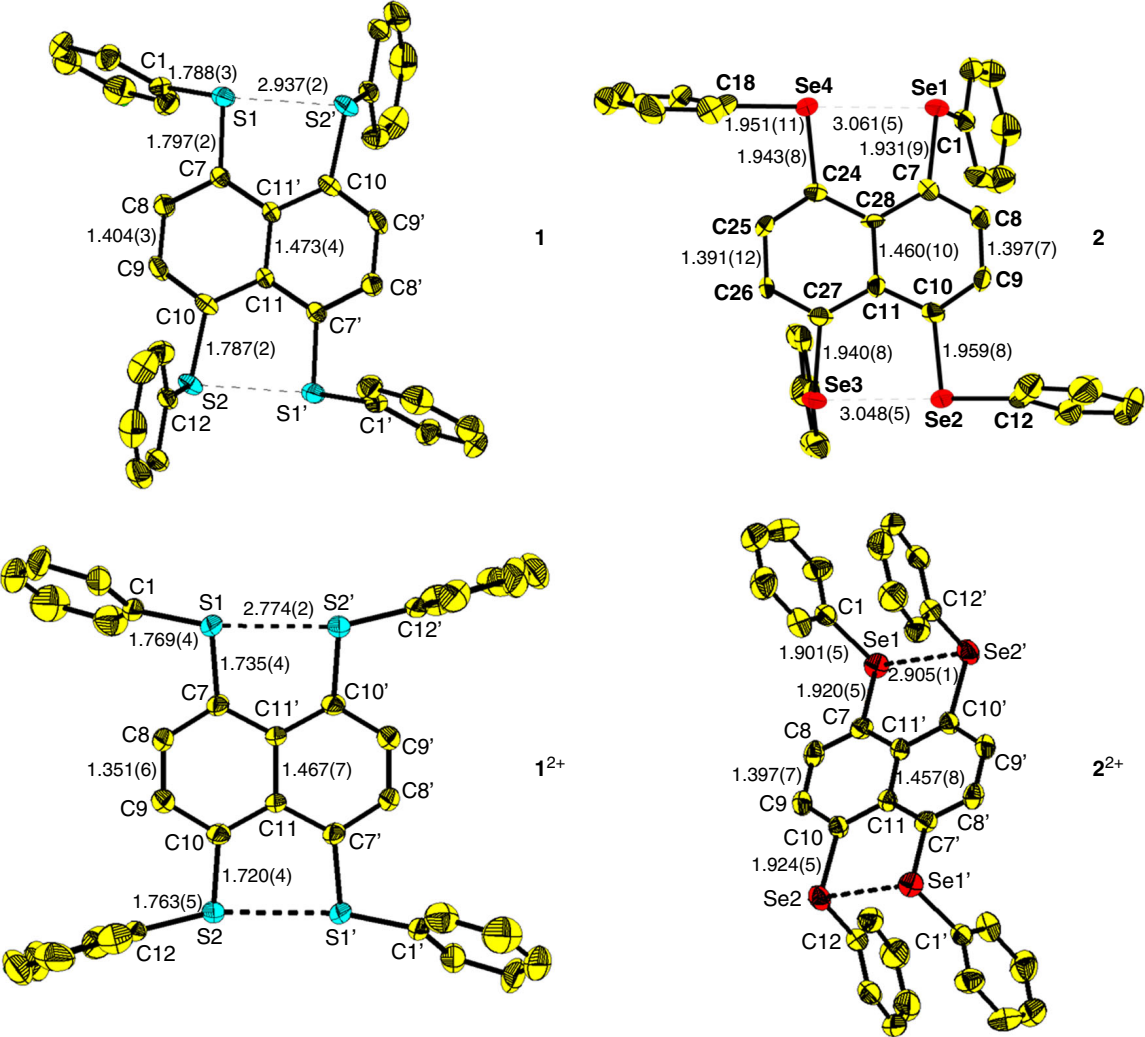
50% ellipsoid drawings of 1, 1^2+^, 2, and 2^2+^. Yellow, carbon; red, selenium; blue, sulfur. Hydrogen atoms are not shown. Selected bond length (Å) and angle (deg): **1** S1^…^S2′ 2.937(2), S1–C1 1.788(3), S1–C7 1.797(2), C7–C8 1.375(3), C8–C9 1.404(3), C9–C10 1.365(4), C10–C11 1.438(3), C11–C11′ 1.473(4), S2–C10 1.787(2), S2–C12 1.787(3), C1–S1–C7 102.3(1), C1–S1–S2′ 163.3(7), C10–S2–C12 102.4(1), C12–S2–S1′ 83.2(7); **1**^2+^ S1–S2′ 2.774(2), S1–C1 1.769(4), S1–C7 1.735(4), C7–C8 1.412(6), C8–C9 1.351(6), C9–C10 1.418(6), C10–C11 1.426(6), C11–C11′ 1.467(7), S2–C10 1.720(4), S2–C12 1.763(5), C1–S1–C7 105.0(2), C1–S1–S2′ 164.5(2), C10–S2–C12 105.3(2), C12–S2–S1′ 161.8(2); **2** Se1^…^Se4 3.061(5), Se2^…^Se3 3.048(5), Se1–C1 1.917(10), Se1–C7 1.931(9), C7–C8 1.349(13), C8–C9 1.388(13), C9–C10 1.338(12), C10–C11 1.439(12), C11–C28 1.460(10), C7–C28 1.436(12), Se2–C10 1.959(8), Se2–C12 1.934(9), C1–Se1–C7 99.4(4), C1–Se1–Se4 124.4(6), C10–Se2–C12 98.2(4), C12–Se2–Se3 175.9(2); **2**^2+^ Se1–Se2′ 2.905(1), Se1–C1 1.901(5), Se1–C7 1.920(5), C7–C8 1.362(7), C8–C9 1.397(7), C9–C10 1.359(7), C10–C11 1.421(6), C11–C11′ 1.457(8), Se2–C10 1.924(5), Se2–C12 1.908(5), C1–Se1–C7 101.2(2), C1–Se1–Se2′ 102.0(1), C10–Se2–C12 99.2(2), C10–Se2–Se1′ 95.7(1).

Crystals suitable for X-ray crystallographic studies were obtained by cooling solutions of neutral tetrachalcogenides and their oxidized species. Their crystal structures are shown in Fig. 3. Some structural parameters are listed in Table 1. In the molecular geometries of **1** and **2**, one Ch–C_Ph_ (Ch = S, Se) bond is nearly perpendicular to the other at both sides of the naphthalene skeleton. Upon oxidation, in **1**^2+^ the two S–C_Ph_ bonds at the same side of the naphthalene skeleton are nearly linear (torsion angle ∠C_Ph_SSC_Ph_ = 6°) and all four S–C_Ph_ bonds are coplanar to the naphthalene skeleton, while in **2**^2+^ two Se–C_Ph_ bonds at the same side are parallel and all Se–C_Ph_ bonds are nearly perpendicular to the naphthyl plane (∠CSeC = 100°). The average Se–C bond (Se–C_Ph_ and Se–C_Nap_) lengths in **2**^2+^ are slightly shorter while ∠C–Se–C angles are slightly larger than those in neutral **2**. The Se•••Se separation (2.905(1) Å) is shorter than that (3.054(2) Å) in **2**, but longer than the Se–Se single bond length (ca. 2.34 Å)^40^. The Se–C_Ph_ bond alignment and structural parameters of **2**^2+^ are similar to those of (NapSe_2_Ph_2_)^+^^30^, indicating there is a three-electron σ-bond between two Se atoms, and the whole dication possesses two three-electron σ-bonds. In contrast, though the S•••S separation (2.774(2) Å) is also shorter than that (2.937(2) Å) in **1**, it is much longer than a regular S–S single bond (ca. 2.05 Å) and comparable to those of molecules with weak intramolecular S•••S interactions^41^. The S–C_nap_ bond length (1.727(4) Å) of **1**^2+^ is also notably shorter than those of **1** (1.792(2) Å) and (NapS_2_Ph_2_)^+^ (1.768(3) Å)^30^. Moreover, the naphthalene skeleton of **1**^2+^ becomes quinoidal (C8–C9 1.351(6) Å).

**Table 1:** Comparison of structural parameters (average) of neutral and dications.


	1 (X-ray)	1 (DFT)	1^2+^ (X-ray)	1^2+^-cs (DFT)	3^2+^-cs (DFT)
S^…^S, Å	2.937(2)	3.025	2.774(2)	2.859	2.868
S–C_Ph_, Å	1.787(3)	1.799	1.766(4)	1.787	1.796
S–C_Nap_, Å	1.792(2)	1.801	1.727(4)	1.750	1.754
∠C–S–C, deg	102.3(1)	103.1	105.2(2)	105.8	105.9
∠S–S–C_Ph_, deg	163.3(1) 83.2(1)	173.7 94.0	163.1(2)	149.6	169.2
	**2** (X-ray)	**2** (DFT)	**2**^2+^ (X-ray)	**2**^2+^-os (DFT)	
Se^…^Se, Å	3.054(5)	3.103	2.905(1)	2.971	2.928
Se–C_Ph_, Å	1.935(8)	1.946	1.910(7)	1.914	1.916
Se–C_Nap_, Å	1.945(9)	1.94	1.917(6)	1.936	1.890
∠C–Se–C, deg	98.6(3)	97.8	100.2(1)	101.9	101.8
∠Se–Se–C_Ph_, deg	118.1(3)	113.2	98.8(1)	105.9	138.2
	176.9(3)	168.6			

### Spectroscopic characterization and SQUID measurements

These dications were further characterized by UV absorption spectroscopy, EPR spectroscopy, and SQUID measurements. The UV–Vis absorption spectra of **1**^**2+**^•2[Al(OR_F_)_4_]^−^ and **2**^**2+**^•2[Al(OR_F_)_4_]^−^ solutions show characteristic absorptions at 710 and 610 nm, respectively, (Supplementary Figs. 2 and 4).

**Fig. 4:**
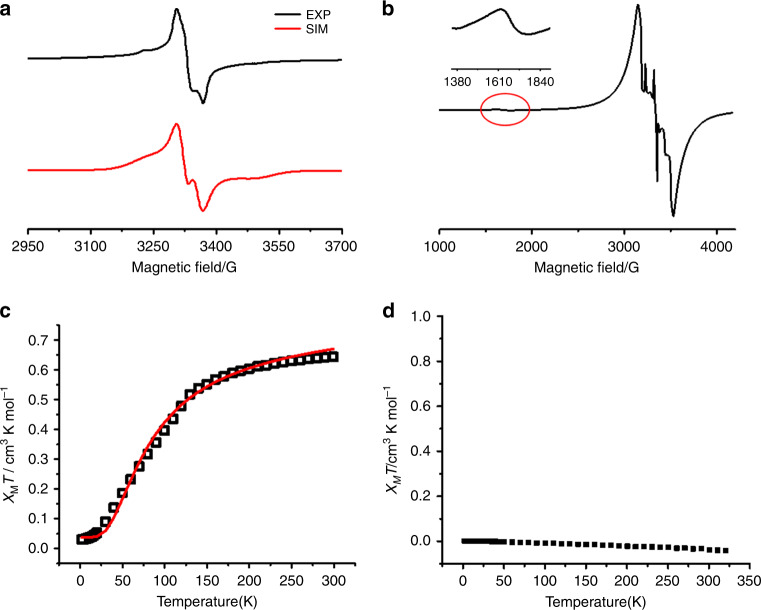
EPR spectra and temperature-dependent plots of *χ*_*M*_*T* for the crystals of 2^2+^. **a** The EPR spectrum of frozen solution of **2**^2+^ (1 × 10^−4^ mol/l) at 183 K (in black) with simulation (in red). **b** The EPR spectrum of the powder sample of **2**^2+^ at 183 K with the forbidden transition at the half magnetic field. **c** Temperature-dependent plots of *χ*_*M*_*T* for the crystals of **2**^**2+**^ from 2 to 320 K (in black) with the fitting plot via the Bleaney–Bowers equation (in red). **d** Temperature-dependent plots of *χ*_*M*_*T* for the crystals of **1**^**2+**^ from 2 to 320 K.

The EPR spectrum (Fig. 4a) of the frozen solution of **2**^2+^•2[Al(OR_F_)_4_]^−^ appears typical of a triplet state with the zero-field parameters *D* (136.0 G), *E* (53.0 G) and an anisotropic *g* factor (*g*_x_ = 2.0190, *g*_y_ = 2.0250, and *g*_z_ = 2.0020) determined by spectral simulation. The *g*_iso_ value (2.0153) is slightly smaller than that of (NapSe_2_Ph_2_)^**•+**^ (2.0236)^29^. The average spin–spin distance was estimated to be 5.9 Å from the *D* parameter, which is comparable to the distance (6.6 Å) between the middle points of Se^…^Se bonds in the X-ray structure. The forbidden Δ*m*_s_ = ±2 transition was not observed from the frozen solution due to the low spin concentration, but observed at the half region of the EPR spectrum on the powder sample of **2**^2+^•2[Al(OR_F_)_4_]^−^ (Fig. 4b), indicating that **2**^2+^ is a diradical dication. An increasing susceptibility with temperature was observed for the powder sample of **2**^2+^ (Fig. 4c). Careful fitting with Bleaney–Bowers equation^42^ gave a singlet–triplet energy gap (Δ*E*_S–T_ = −0.29 kcal mol^−1^), confirming that **2**^2+^ has an open-shell singlet (OS) ground state. To exclude the intermolecular electronic interaction, frozen solution variable-temperature EPR spectroscopy was performed (Supplementary Fig. 5). *AT* is the product of the intensity for the Δ*m*_*s*_ = 2 resonance and the temperature (*T*)^43^.

The plot of ln(*AT*) versus 1/*T* gives the singlet–triplet gap Δ*E*_S–T_ of −0.14 kcal mol^−1^, which is close to that obtained from SQUID measurement, further confirming the intra-antiferromagnetic interaction. In contrast, both the frozen solution and powder samples of **1**^2+^ are EPR silent, which together with the diamagnetism observed by SQUID measurement (Fig. 4d) indicates **1**^2+^ has a closed-shell structure in the ground state.

### Theoretical calculations

To explore their electronic structures, we performed density functional theory (DFT) calculations on neutral molecules and dications. We first used the crystal structure of **2**^**2+**^ and **1**^2+^ as the starting geometries for optimization of their close-shell singlets (CS), open-shell singlets (OS), and triplets at the (U)B3LYP/6-31+G(d,p) level. **2**^2+^ has an OS ground state (**2**^2+^-os) while **1**^2+^ has a closed-shell singlet ground state (**1**^2+^-cs) (Supplementary Table 2). The closed-shell state of **2**^2+^ (**2**^2+^-cs) has a similar geometry to that of **1**^2+^-cs. However, a geometry optimization starting with **2**^2+^-cs does not reach **2**^2+^-os, probably due to a high energy barrier. The optimized geometries of these dications with the lowest energy reasonably agree with the X-ray crystal structures (Table 1). A hypothetical mixed dication species **3**^2+^ with two S atoms and two Se atoms at each side of the naphthalene skeleton was also computed (Table 1), which has a closed-shell singlet ground state with the geometry similar to that of **1**^2+^. However, the energy difference between the closed-shell singlet and the triplet is lower than that of **1**^2+^ but higher than that of **2**^2+^, showing the atom dependence (Supplementary Table 2).

**Fig. 5:**
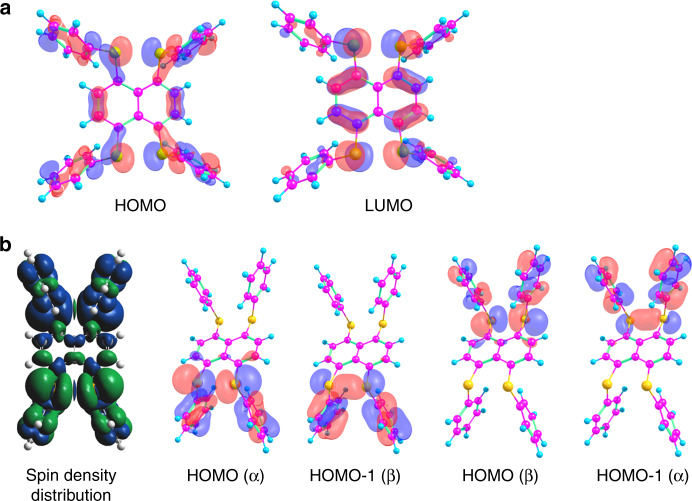
Molecular orbitals and spin density distribution. **a** Frontier molecular orbitals of **1**^2+^. **b** The spin density distribution and some molecular orbitals of **2**^2+^.

**Fig. 6:**
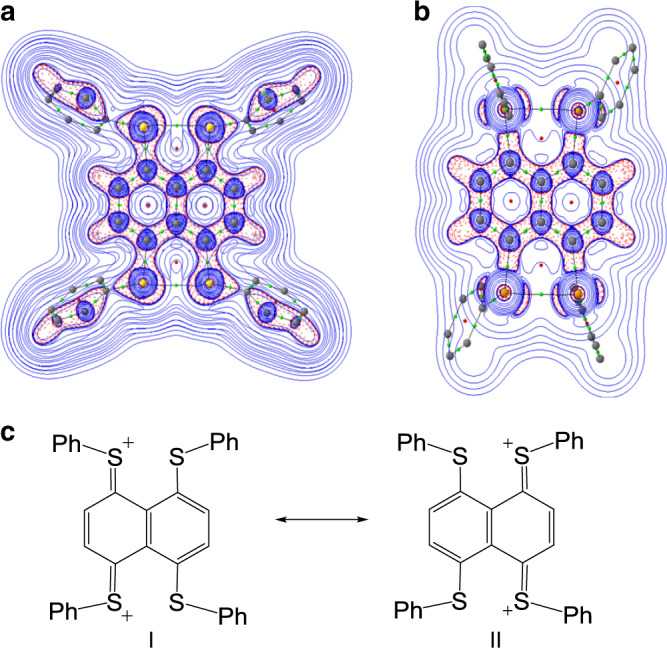
Plots of the Laplacian ∇^2^*ρ*(r) and resonance structures. Plots of the Laplacian ∇^2^*ρ*(r) for **1**^**2+**^ (**a**) and **2**^**2+**^ (**b**). Red dashed lines indicate areas of charge concentration (∇^2^*ρ*(r) < 0), while solid blue lines show areas of charge depletion (∇^2^*ρ*(r) > 0). The solid lines connecting the atomic nuclei are the bond paths. Green dots are bond critical points and red dots are ring critical points. **c** Resonance structures of **1**^**2+**^.

Consistent with the experimental data, all four S–C_ph_ bonds in **1**^2+^-cs are nearly coplanar with the naphthalene skeleton, leading to a quinoidal geometry reflected by the HOMO (Fig. 5a). The decrease of the S•••S separation from **1** to **1**^2+^ indicates considerable intramolecular S•••S interaction^40^, which is supported by Wiberg bond order of S–S bond (0.19). In **2**^2+^-os, the spin density is mainly on Se atoms with an additional extension to the four phenyl rings and naphthalene skeleton (Fig. 5b). The calculated Wiberg bond order for two Se–Se bonds (0.43, 0.43), together with calculated Se–Se antibonding and bonding orbitals (Fig. 5b), indicates the formation of a 2c–3e hemi bond between Se atoms at both sides of the naphthalene skeleton. The calculated miniscule singlet–triplet energy gap (−0.20 kcal mol^−1^) is in agreement with the value determined from SQUID measurement. Figure 6 shows the Laplacian distribution ∇^2^*ρ*(r), the bond paths and critical points of **1**^**2+**^ and **2**^**2+**^ in a plane that contains Ch (Ch = S, Se) atoms and the naphthalene skeleton. It clearly shows the S–S and Se–Se bonding character, as indicted by the bond critical point between the Ch–Ch centers. Judging from the time-dependent DFT (TD-DFT) calculations (Supplementary Figs. 2 and 4), the UV absorptions are mainly assigned to HOMO→LUMO (for **1**^2+^) and HOMO-1 (α/β)→LUMO (α/β) (for **2**^**2+**^), respectively, (Fig. 5).

Since it is a complicated system to perform complete-active-space SCF (CASSCF) calculation that will take a large active space, we performed a CAS(2,4) calculation with B3LYP optimized geometry to check whether we can call confidently that **2**^**2+**^ possesses an OS state. The resulting Löwdin natural orbitals (NOs) derived from the CASSCF density matrix and their occupation is given in Supplementary Fig. 6. The corresponding occupation number and the shape of the NOs corroborate with the DFT finding.

## Discussion

We, here, have shown that tetrachalcogenides **1** and **2** with a naphthalene bridge underwent two-electron oxidations, which afforded room temperature stable dications **1**^2+^ and **2**^2+^. **1**^2+^ is shown to possess a closed-shell singlet ground state while **2**^2+^ is a diradical containing two Se∴Se three-electron σ-bonds with the electronic coupling of −0.29 kcal mol^−1^. The difference of electronic structures between two dications **1**^**2+**^ and **2**^2+^ is attributed to the easier p_π_–p_π_ interaction between sulfur and carbon atoms than that between selenium and carbon atoms in terms of atomic size matching. The experimentally obtained geometry of **1**^**2+**^ may be rationalized by that two sulfur atoms from each side of the molecule loses one electron and form a quinoidal structure (I and II, Fig. 6c) with a 14c–16e π-bond upon two-electron oxidation. Though the S•••S separation was observed from **1** to **1**^**2+**^ decreases, the S–S bond length in **1**^**2+**^ (2.77 Å) is much longer than a typical S–S single bond (2.05 Å)^41^. Thus the geometry of **1**^**2+**^ is best described as the hybrid of resonance structures of I and II, which is supported by the calculated HOMO (Fig. 5a). In contrast, the difficult formation of p_π_–p_π_ bonding between selenium and carbon atoms makes dication **2**^2+^ as a diradical containing two Se∴Se three-electron σ-bonds. **2**^2+^ represents the first example of a diradical based on odd-electron σ-bonds. The hypothetical mixed singlet species **3**^**2+**^ has a similar quinoidal geometry as **1**^**2+**^, which may also be induced by sulfur and carbon p_π_–p_π_ interaction. The work sheds new light on the concepts of both diradicals and odd-electron bonds. Synthesis of more diradicals based on odd-electron bonds is under way in our laboratory.

## Methods

### General

All manipulations were carried out under an N_2_ atmosphere by using standard Schlenk or glove box techniques. Solvents were dried prior to use. 1,4,5,8-tetrabromo naphthalene^38^ and Li[Al(OR_F_)_4_] (OR_F_ = OC(CF_3_)_3_)^39^ were synthesized according to the literature procedures. NOSbF_6_, diphenyldisulfane (PhSSPh), diphenyldiselane (PhSeSePh), isochromeno[6,5,4-def]isochromene-1,3,6,8-tetraone, and ^*n*^BuLi (1.60 M, in hexane) were purchased from Energy Chemical. CV was performed on an IM6ex electrochemical workstation, with platinum as the working and counter electrodes, Ag/Ag^+^ as the reference electrode and 0.2 M ^*n*^Bu_4_NPF_6_ as the supporting electrolyte. The NMR spectra were performed using a Bruker DRX-400 at room temperature in ppm downfield from internal Me_4_Si. EPR spectra were obtained using Bruker EMX-10/12 X-band variable-temperature apparatus. UV–Vis spectra were recorded on the Lambda 750 spectrometer. Element analyses of **1**^2+^•2[Al(OR_F_)_4_]^−^ and **2**^2+^•2[Al(OR_F_)_4_]^−^ were performed at Shanghai Institute of Organic Chemistry, the Chinese Academy of Sciences. Magnetic measurements were performed using a Quantum Design SQUID VSM magnetometer with a field of 0.1 T. X-ray crystal structures were obtained by using Bruker D8 CMOS detector at 193 K. Crystal data and structure refinement are listed in Supplementary Table 1.

*Preparation of (4,8-dibromonaphthalene-1,5-diyl)bis(phenylsulfane)*: A solution of ^*n*^BuLi (6.90 ml, 1.60 M, 11.04 mmol) in hexane was added dropwise to a solution of 1,4,5,8-tetrabromo naphthalene (2.24 g, 5.05 mmol) in Et_2_O (150 ml) at −78 °C and stirring was maintained for 2 h. Then a solution of PhSSPh (2.40 g, 11.00 mmol) in Et_2_O (20 ml) was added dropwise to the mixture. Then the resulting mixture was allowed to reach to room temperature and stirring was continued for 12 h. The crude product was treated with 0.10 M solution of sodium hydroxide (3 × 30 ml) and extracted with Et_2_O. The combined organic phase was dried over Na_2_SO_4_ and concentrated under vacuum. The crude product was purified by chromatography using petroleum ether: CH_2_Cl_2_ (10: 1) as the eluent to give 1.00 g of (4,8-dibromonaphthalene-1,5-diyl)bis(phenylsulfane) (40%) as light yellow solid. ^1^H NMR(400 MHz, CD_2_Cl_2_) *δ* 7.14 (d, ^3^*J*(H, H) = 8.1 Hz, 2H, Ar-*H*), 7.26–7.32 (m, 10 H, Ar-*H*) 7.60 (d, ^3^*J*(H, H) = 8.1 Hz, 2H, Ar-*H*); ^13^C NMR(125 MHz, CD_2_Cl_2_) *δ* 118.21, 128.24, 129.96, 132.89, 134.32, 134.35, 134.62, 137.09, and 137.36.

*Preparation of (4,8-dibromonaphthalene-1,5-diyl)bis(phenylselane)*: By the procedure similar to the synthesis of the (4,8-dibromonaphthalene-1,5-diyl)bis(phenylsulfane), a yellow solid is given. Yield: 1.01 g, 34.5%; ^1^H NMR (400 MHz, CD_2_Cl_2_) *δ* 7.10 (d, ^3^*J*(H, H) = 8.16 Hz, 2H, Ar-*H*), 7.35–7.41 (m, 6H, Ar-*H*) 7.48 (d, ^3^*J*(H, H) = 8.15 Hz, 2H, Ar-*H*), 7.56–7.58 (m, 4H, Ar-*H*); ^13^C NMR(125 MHz, CD_2_Cl_2_) *δ* 118.75, 129.32, 130.27, 132.34, 133.30, 133.52, 135.13, 135.72, and 136.59.

*Preparation of* ***1***: A solution of ^*n*^BuLi (2.60 ml, 1.60 M, 4.16 mmol) in hexane was added dropwise to a solution of (4,8-dibromonaphthalene-1,5-diyl)bis(phenylsulfane) (1.00 g, 1.99 mmol) in Et_2_O (120 ml) at −78 °C and maintained stirring for 2 h. Then a solution of PhSSPh (0.92 g, 4.21 mmol) in Et_2_O (20 ml) was added dropwise to the mixture. Then the resulting mixture was raised to room temperature and kept stirring for 12 h. The crude product was treated with 0.10 M solution of sodium hydroxide (3 × 30 ml) and extracted with Et_2_O. The combined organic phase was dried over Na_2_SO_4_ and concentrated under vacuum. The crude product was purified by chromatography using petroleum ether: CH_2_Cl_2_ (5: 1) as the eluent to give 0.45 g (0.80 mmol) of **1** (40%) as a dark yellow solid. ^1^H NMR (400 MHz, CD_2_Cl_2_) *δ* 7.17–7.18 (m, 2H, Ar-H), 7.20 (d, ^3^*J*(H, H) = 1.7 Hz, 4H, Ar-*H*), 7.21–7.23 (m, 2H, Ar-H), 7.24–7.25 (m, 2H, Ar-H), 7.26–7.28 (m, 8H, Ar-H), 7.29–7.30 (m, 6H, Ar-H); ^13^C NMR (125 MHz, CD_2_Cl_2_) *δ* 127.49, 129.65, 131.31, 133.68, 135.07, 136.37, and 138.57.

*Preparation of* ***2***: By the procedure similar to the synthesis of **1**, a yellow solid is given. Yield: 0.46 g, 30%; ^1^H NMR (400 MHz, CD_2_Cl_2_) *δ* 7.24–7.25 (m, 2H, Ar-*H*), 7.26–7.27 (m, 4H, Ar-*H*), 7.28–7.30 (m, 6H, Ar-*H*), 7.35–7.36 (m, 4H, Ar-*H*), 7.37–7.38 (m, 4H, Ar-*H*), 7.48 (d, ^3^*J*(H, H) = 1.05 Hz, 4H, Ar-*H*); ^13^C NMR (125 MHz, CD_2_Cl_2_) *δ* 127.96, 129.77, 132.73, 133.31, 135.69, 135.95, and 138.64.

*Preparation of* ***1***^**2**+^•2[Al(OR_F_)_4_]^−^: Under anaerobic and anhydrous conditions, CH_2_Cl_2_ (35 ml) was added dropwise to the mixture of **1** (0.11 g, 0.20 mmol), NOSbF_6_ (0.11 g, 0.42 mmol) and Li[Al(OR_F_)_4_] (0.41 g, 0.42 mmol) while stirring at room temperature. The resultant dark blue solution was stirred at room temperature for 12 h, and then filtered to remove the precipitate (LiSbF_6_). The filtrate was concentrated and stored at −40 °C for 24 h to afford yellow X-ray-quality crystals of **1**^2+^•2[Al(OR_F_)_4_]^−^. Isolated yield: 0.12 g, 24%; elemental analysis (calcd. found for C_66_H_24_Al_2_F_72_O_8_S_4_): C (31.77, 31.48) H (0.97, H 1.14).

*Preparation of* ***2***^2+^•2[Al(OR_F_)_4_]^−^: By the procedure similar to the synthesis of **1**^**2+**^•2[Al(OR_F_)_4_]^−^, black crystals are given. Isolated yield: 0.10 g, 19%; elemental analysis (calcd found for C_66_H_24_Al_2_F_72_O_8_Se_4_): C (29.55, 29.16) H (0.90, H 1.13)

### Quantum chemical calculations

Geometry optimization without symmetry constraint were performed using DFT at the (U)B3LYP/6-31+G(d, p) level. Frequency results were examined to confirm stationary points as minima (no imaginary frequencies). The UV–Vis absorption spectrum was calculated on the optimized geometry using TD-DFT method at the (U)B3LYP/6-31+G(d,p) level. To consider solvent (CH_2_Cl_2_) effects, polarized continuum model was adopted in the calculation of the single point energies involved in the disproportionation and dimerization, and UV–Vis absorption spectrum. Wiberg bond order was calculated at the (U)B3LYP/6-31+G(d,p) level with the Multiwfn program. These calculations were performed using Gaussian 16 A03 software. The electron density distribution was analyzed with Quantum Theory of Atom in Molecules method that was developed by Bader^44^. The multiconfigurational CASSCF^45,46^ calculations were performed on the (U)B3LYP/6-31+G(d, p) optimized geometry of **2**^2+^-os with the def2-SVP basis set using ORCA 4.2.0 program^47^.

## Supplementary information

Supplementary Information

## Data Availability

The authors declare that all relevant data supporting the findings of this work are available from the corresponding authors on request. The X-ray crystallographic coordinates for structures reported in this study have been deposited at the Cambridge Crystallographic Data Centre (CCDC), under deposition numbers 1959358-1959361. These data can be obtained free of charge from The Cambridge Crystallographic Data Centre via www.ccdc.cam.ac.uk/data_request/cif.
