# Supplementary material for: A diradical based on odd-electron σ-bonds
Source: Nat Commun. 2020 Jul 10;11:3441. doi: 10.1038/s41467-020-17303-4 (PMC7351710; doi:10.1038/s41467-020-17303-4)
Supplement: Supplementary file 1 — Supplementary Information [file 41467_2020_17303_MOESM1_ESM.pdf]

Supplementary Information

A Diradical Based on Odd-Electron σ-Bonds

Xinping Wang, et al.

Content

Supplementary Table 1 Crystal data and structure refinement……………………………….S3

Supplementary Figure 1 Absorption spectrum of 1.0 x 10^{-4} M **1** in CH_2Cl_2 at 25 ^{o}C (top) and calculated absorption spectrum of **1** (bottom), together with related molecular orbitals…………………………………………………………………………………………….S4

Supplementary Figure 2 Absorption spectrum of 1.0 x 10^{-4} M $\mathbf{1^{2+}}$ in CH_2Cl_2 at 25 ^{o}C (top) and calculated absorption spectrum of $\mathbf{1^{2+}}$ (bottom), together with related molecular orbitals………S5

Supplementary Figure 3 Absorption spectrum of 1.0 x 10^{-4} M **2** in CH_2Cl_2 at 25 ^{o}C (top) and calculated absorption spectrum of **2** (bottom), together with related molecular orbitals…………………………………………………………………………………………….S6

Supplementary Figure 4 Absorption spectrum of 1.0 x 10^{-4} M $\mathbf{2^{2+}}$ in CH_2Cl_2 at 25 ^{o}C (top) and calculated absorption spectrum of $\mathbf{2^{2+}}$ (bottom), together with related molecular orbitals…………………………………………………………………………………………….S7

Supplementary Figure 5 | Plot of ln(*AT*) versus 1/*T* of the frozen solution sample of $\mathbf{2^{2+}}$……....S8

Supplementary Table 2 | Energy difference $(\Delta E = E_X - E_{CS})$ ……………………………….S9

Supplementary Figure 6 | The Löwdin natural orbitals (NOs) and their occupation derived from the CASSCF density matrix…………………………………………………………………….S9

Computational details……………………………………………………………………..S10

Coordinates………………………………………………………………………………S10

Supplementary References………………………………………………………………S29

Supplementary Table 1 | Crystal data and structure refinement

	**1**	$\mathbf{1}^{2+}$•2[Al(OR$_F$)$_4^-$]	**2**	$\mathbf{2}^{2+}$•2[Al(OR$_F$)$_4^-$]
Formula	$C_{17}H_{12}S_2$	$C_{33}H_{12}AlF_{36}O_4S_2$	$C_{34}H_{24}Se_4$	$C_{33}H_{12}AlF_{36}O_4Se_2$
M/g mol^{-1}	280.39	1247.53	748.37	1341.33
Crystal system	Triclinic	Triclinic	Triclinic	Triclinic
Space group	*P*-1	*P* -1	*P* -1	*P* -1
a, Å	5.7779(4)	11.6485(12)	10.041(3)	10.7793(7)
b, Å	9.4401(7)	18.681(2)	13.988(4)	15.9741(11)
c, Å	12.6127(10)	20.033(2)	15.243(4)	25.7523(16)
α, deg	96.089(2)	90.601(4)	81.046(5)	88.343(2)
β, deg	92.815(2)	91.945(4)	88.113(4)	86.395(2)
γ, deg	95.838(2)	91.723(3)	86.341(4)	78.114(2)
V, Å^3	679.27(9)	4354.5(8)	2110.0(10)	4330.0(5)
Z	2	4	3	4
Temperature, K	193(2)	120(2)	193(2)	153(2)
*R*1 (I>2σ(I))	0.0372	0.1434	0.0767	0.0954
*wR*2 (all data)	0.0832	0.2357	0.1879	0.2138

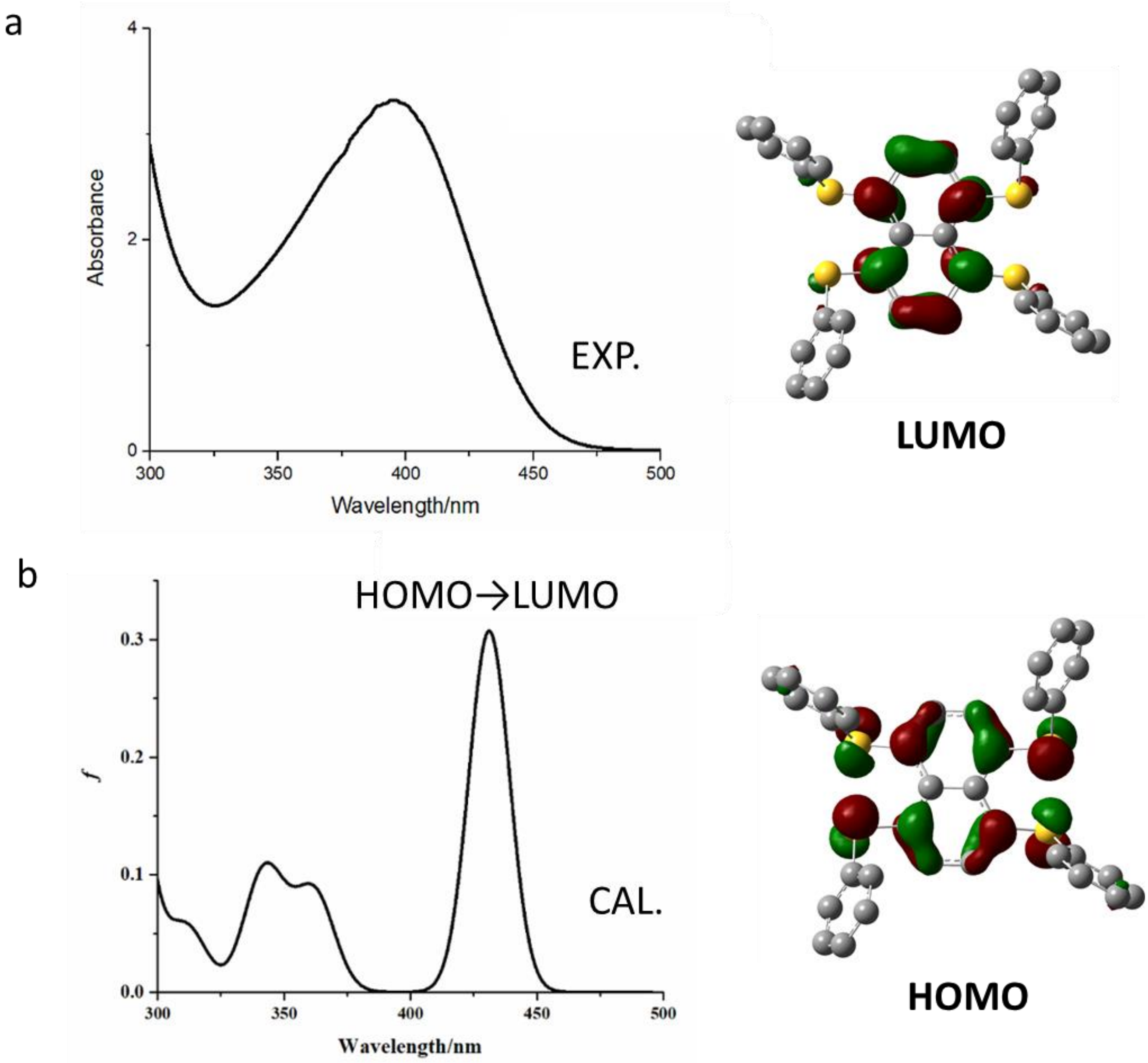

Supplementary Figure 1 | Absorption spectra and related molecular orbitals of 1. a. Absorption spectrum CH_2Cl_2 (1.0 x 10^{-4} M) at 25 ^oC; **b.** Calculated absorption spectrum.

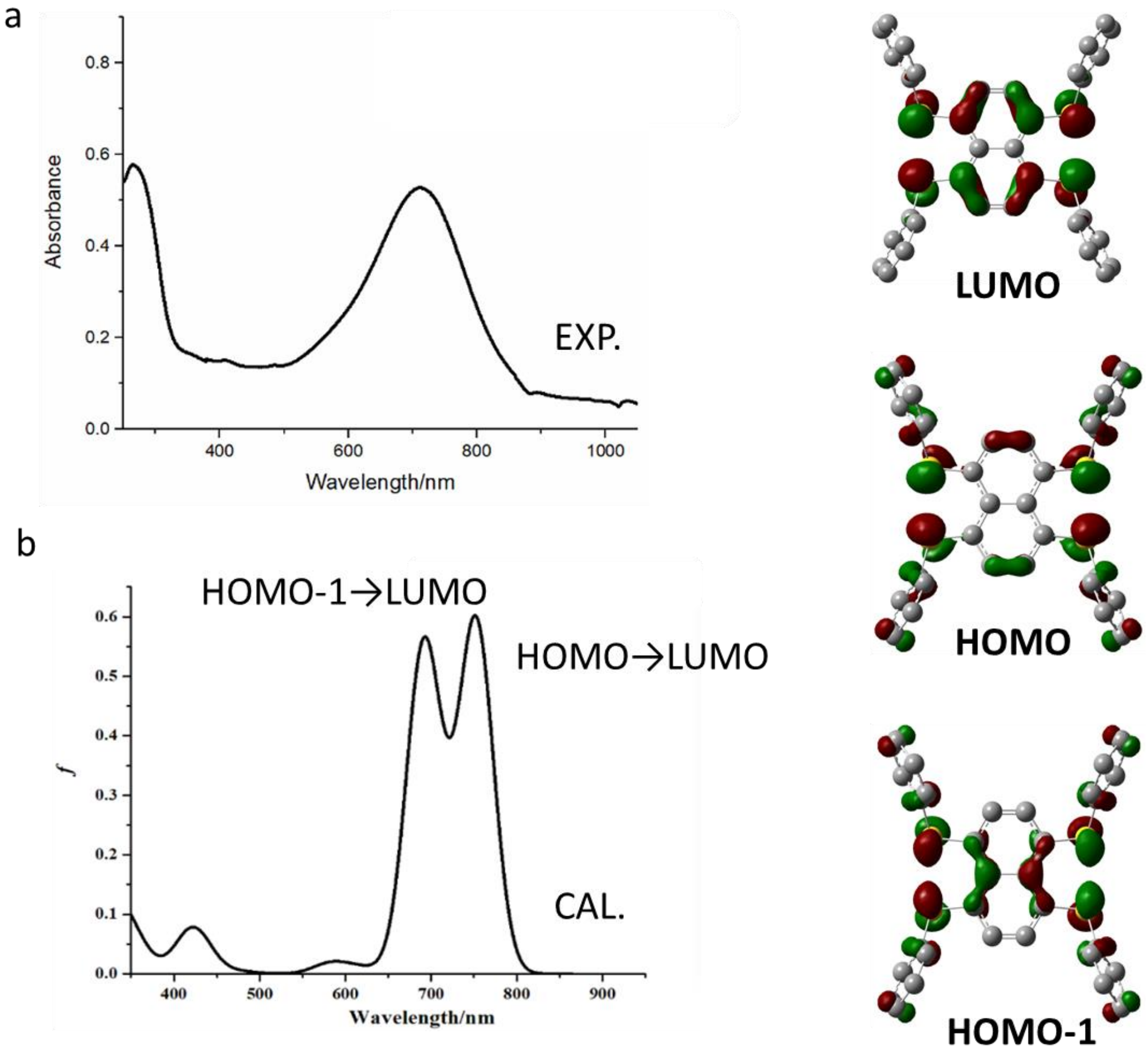

Supplementary Figure 2 | Absorption spectra and related molecular orbitals of 1^{2+}. a. Absorption spectrum CH_2Cl_2 (1.0 x 10^{-4} M) at 25 ^{o}C; **b.** Calculated absorption spectrum.

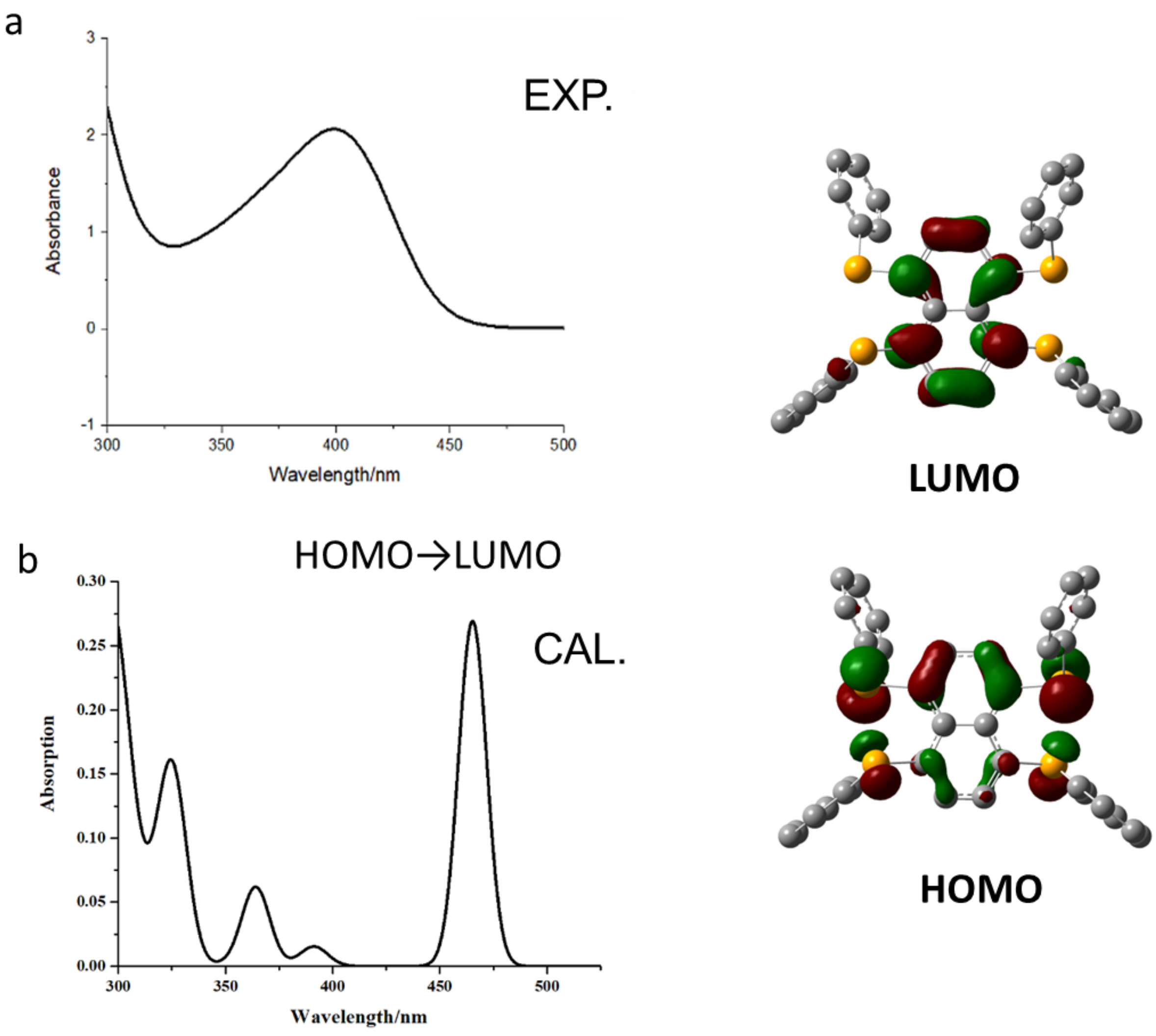

Supplementary Figure 3 | Absorption spectra and related molecular orbitals of 2. a. Absorption spectrum CH_2Cl_2 (1.0 x 10^{-4} M) at 25 ^oC; **b.** Calculated absorption spectrum.

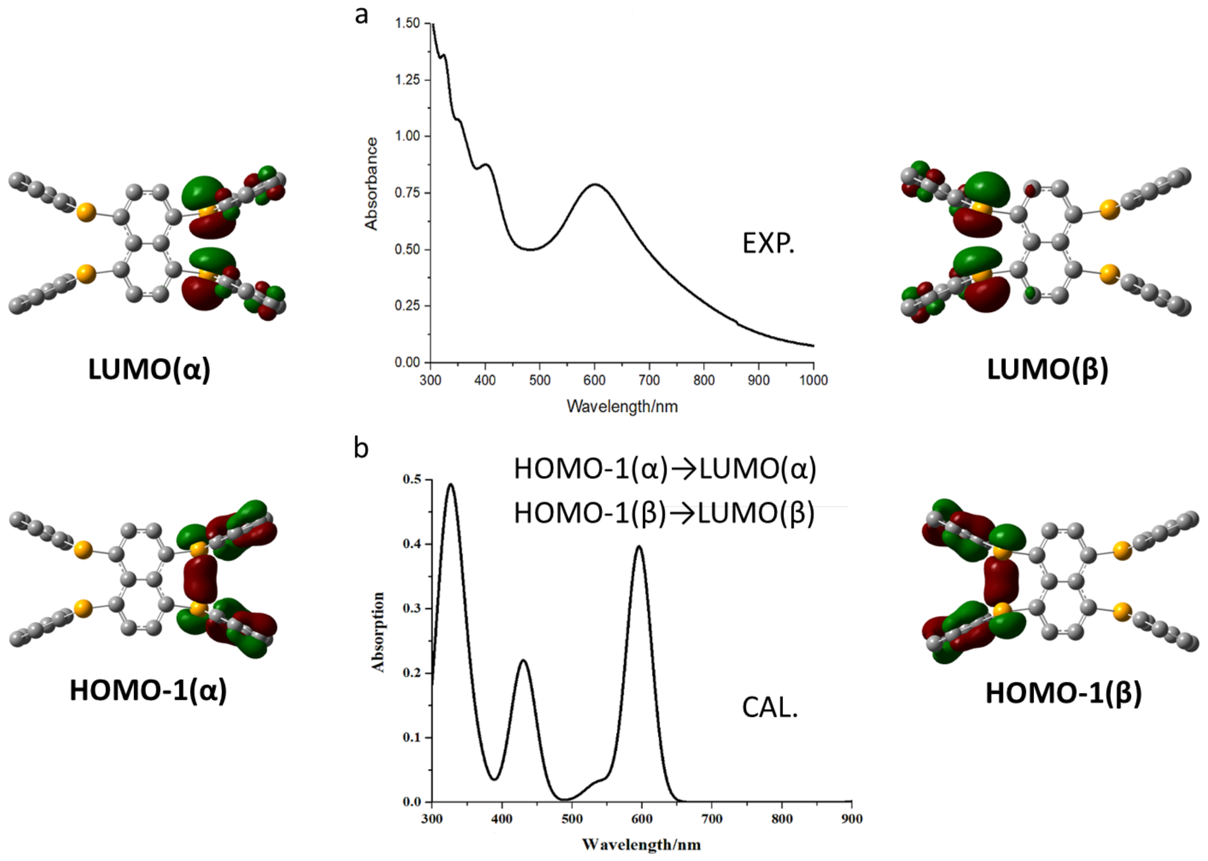

Supplementary Figure 4 | Absorption spectra and related molecular orbitals of 2^{2+}. a. Absorption spectrum CH_2Cl_2 (1.0 x 10^{-4} M) at 25 ^{o}C; **b.** Calculated absorption spectrum.

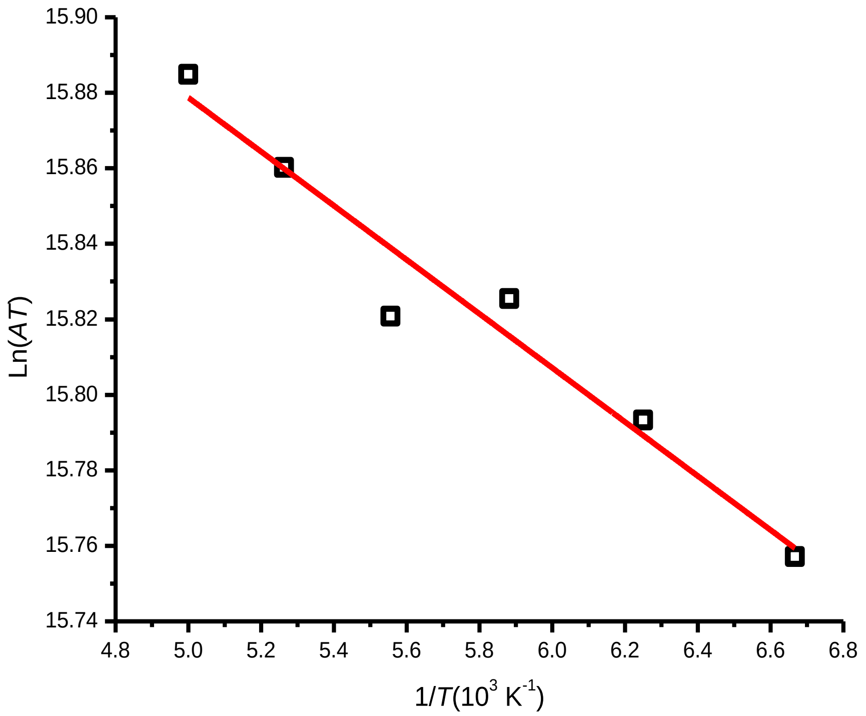

Supplementary Figure 5 | Plot of ln(*AT*) versus 1/*T* based on variable-temperature EPR spectra of the frozen solution sample of 1.0 x 10^{-4} M 2^{2+}. *AT* is the product of the intensity (*A*) for the Δm_s = 2 resonance and the temperature (*T*).

Supplementary Table 2 | Energy difference $(\Delta E = E_X - E_{CS}, \mathrm{kcal/mol})$ of **1**$^{2+}$, **2**$^{2+}$ and **3**$^{2+}$ at (U)B3LYP/6-31+G(d,p) level

	**1**$^{2+}$	**2**$^{2+}$	**3**$^{2+}$
CS[a]	0	0	0
OS[a]	/[b]	-2.95	/[b]
T[a]	7.49	-2.75	2.54

a. CS: close-shell singet, OS: open-shell singlet, T: triplet; b. OS of **1**$^{2+}$ and **3**$^{2+}$ cannot be found and the stationary points finally went to the closed-shell singlets.

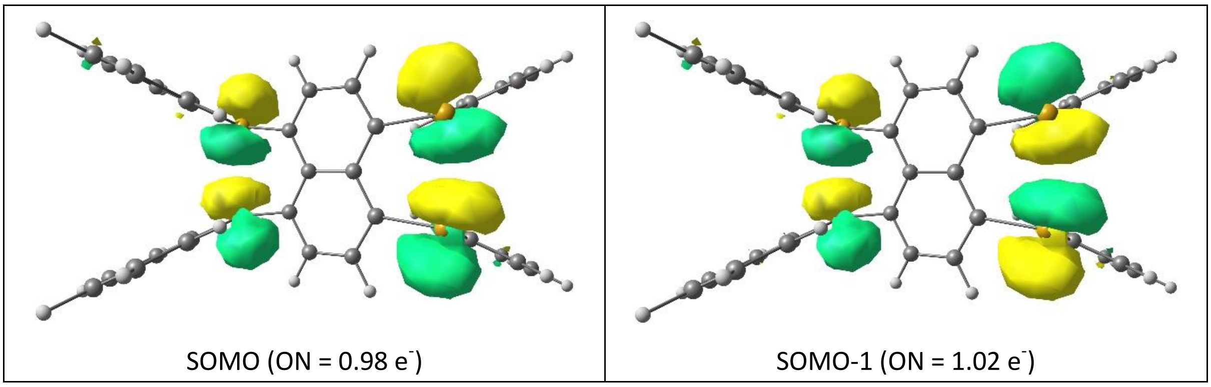

Supplementary Figure 6 | The Löwdin natural orbitals (NOs) and their occupation derived from the CASSCF density matrix.

Supplementary Methods

Computational details:

Geometry optimization without symmetry constraint were performed with DFT (density functional theory) method by using the B3LYP[S1] functional and the Ahlrichs 6-31+G(d, p)[S2] basis function. Frequency results were examined to confirm stationary points as minima (no imaginary frequencies). The molecular orbitals were calculated at the level of (U)B3LYP/6-31+G(d,p) on the optimized geometries. The UV-vis absorption spectra were calculated using the time-dependent DFT (TD-DFT) method at (U)B3LYP/6-31+G(d,p). These calculations were performed using Gaussian 16 A03 software.[S3] We analyzed the electron density distribution at the (U)B3LYP/6-31+G(d, p) level with QTAIM (Quantum Theory of Atom in Molecules) method that was developed by Bader.[S4] The multi-configurational complete-active-space SCF (CASSCF)[S5,S6] calculations were performed on the (U)B3LYP/6-31+G(d, p) optimized geometry of **2**$^{2+}$-os with the def2-SVP basis set using ORCA 4.2.0 program[S7].

Coordinates

1

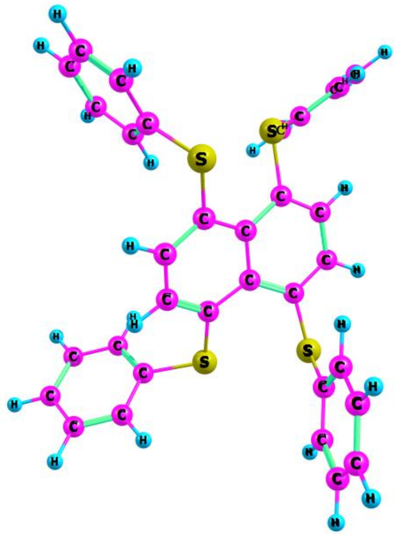

S	-2.618513000	-2.662947000	-0.963983000
S	3.363712000	-0.847399000	0.488478000
C	-0.758633000	-0.645311000	-0.123315000

C	-1.007294000	-2.002212000	-0.539779000
C	1.627131000	-1.283498000	0.223419000
C	-3.468410000	-2.765770000	0.615883000
C	1.344269000	-2.539674000	-0.276884000
H	2.140683000	-3.260246000	-0.418173000
C	0.029788000	-2.912041000	-0.592054000
H	-0.179402000	-3.935879000	-0.883703000
C	4.181532000	-1.856843000	-0.763036000
C	-2.809380000	-2.834822000	1.849772000
H	-1.726571000	-2.789445000	1.894790000
C	5.068771000	-2.858517000	-0.348810000
H	5.179366000	-3.070880000	0.710269000
C	-4.869109000	-2.831173000	0.571836000
H	-5.384144000	-2.760100000	-0.381963000
C	4.041365000	-1.577292000	-2.131195000
H	3.366635000	-0.789832000	-2.452617000
C	-3.550569000	-2.963631000	3.027134000
H	-3.030032000	-3.010805000	3.979619000
C	5.805561000	-3.579595000	-1.295084000
H	6.492793000	-4.353481000	-0.965235000
C	5.652177000	-3.310273000	-2.656249000
H	6.220966000	-3.872595000	-3.390979000
C	-5.599729000	-2.977982000	1.752200000
H	-6.683890000	-3.030810000	1.705802000
C	4.766523000	-2.308809000	-3.071819000
H	4.649774000	-2.089838000	-4.129439000
C	-4.945170000	-3.040423000	2.985799000
H	-5.515337000	-3.145614000	3.903909000
S	2.055321000	1.169034000	2.320026000
S	-3.187762000	0.296440000	-1.277562000
C	0.551690000	-0.339915000	0.443938000
C	0.699450000	0.884674000	1.181075000
C	-1.719145000	0.428473000	-0.230693000
C	2.937241000	2.576782000	1.644975000
C	-1.483157000	1.622479000	0.431254000
H	-2.214551000	2.419508000	0.392516000
C	-0.322598000	1.813890000	1.187565000
H	-0.219231000	2.715404000	1.782730000
C	-3.656305000	2.023435000	-1.499598000

C	2.906830000	2.936615000	0.291828000
H	2.302496000	2.370512000	-0.408249000
C	-2.993833000	2.829114000	-2.437753000
H	-2.138053000	2.432342000	-2.975269000
C	3.730331000	3.310069000	2.540772000
H	3.748664000	3.040967000	3.593287000
C	-4.775648000	2.524688000	-0.821959000
H	-5.291496000	1.895141000	-0.103319000
C	3.657428000	4.027023000	-0.154051000
H	3.624200000	4.300029000	-1.205200000
C	-3.436985000	4.130782000	-2.678074000
H	-2.918560000	4.750714000	-3.403979000
C	-4.551709000	4.631448000	-1.996407000
H	-4.898440000	5.642347000	-2.190100000
C	4.487908000	4.389298000	2.082624000
H	5.098026000	4.949569000	2.785488000
C	-5.221744000	3.826512000	-1.072199000
H	-6.090614000	4.208653000	-0.544042000
C	4.452487000	4.756106000	0.734200000
H	5.036198000	5.600645000	0.380620000

1^{2+}-cs

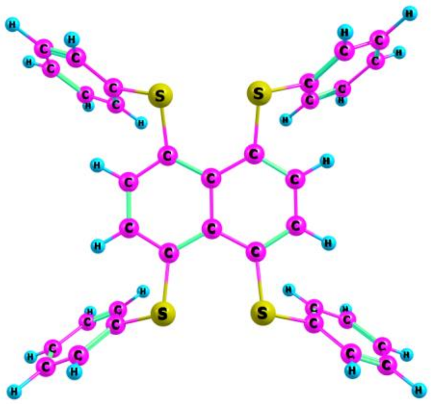

S	3.151081000	1.429594000	-0.855362000
S	3.151297000	-1.429108000	-0.855381000
S	-3.151282000	1.429064000	-0.855386000
S	-3.151067000	-1.429560000	-0.855329000
C	-0.736181000	-0.000058000	-0.679037000
C	-0.682452000	-2.460662000	-0.455657000
C	3.600620000	-2.970280000	-0.070589000
C	0.682842000	-2.460560000	-0.455666000

C	-1.424192000	1.265428000	-0.628496000
C	-1.423997000	-1.265648000	-0.628467000
C	3.600156000	2.970825000	-0.070545000
C	4.805523000	-5.052986000	-0.239786000
C	-3.600153000	-2.970799000	-0.070535000
C	1.424016000	1.265654000	-0.628486000
C	1.424210000	-1.265437000	-0.628483000
C	-4.325705000	-3.890358000	-0.844049000
C	3.863666000	4.354660000	1.887627000
C	-4.573497000	-5.286433000	1.120101000
C	-3.380306000	3.186334000	1.301282000
C	4.325666000	3.890416000	-0.844061000
C	-0.682832000	2.460558000	-0.455697000
C	4.574324000	-5.285772000	1.120028000
C	-3.863635000	-4.354670000	1.887615000
C	-3.379760000	-3.186821000	1.301381000
C	3.864369000	-4.354097000	1.887563000
C	3.379799000	3.186812000	1.301381000
C	4.804737000	5.053720000	-0.239702000
C	-4.804784000	-5.053664000	-0.239701000
C	3.380313000	-3.186318000	1.301338000
C	-4.326295000	3.889684000	-0.844190000
C	0.736208000	0.000056000	-0.679040000
C	4.326264000	-3.889749000	-0.844125000
C	4.573485000	5.286455000	1.120112000
C	0.682462000	2.460666000	-0.455689000
C	-4.805569000	5.052930000	-0.239878000
C	-4.574357000	5.285761000	1.119926000
C	-3.600625000	2.970252000	-0.070636000
C	-3.864376000	4.354121000	1.887479000
H	4.507074000	-3.704734000	-1.898400000
H	5.359919000	-5.774323000	-0.831187000
H	4.957544000	-6.188037000	1.585786000
H	3.704630000	-4.529339000	2.946619000
H	2.857067000	-2.448480000	1.901397000
H	1.207962000	-3.406029000	-0.403417000
H	-1.207431000	-3.406208000	-0.403397000
H	1.207436000	3.406215000	-0.403444000
H	-1.207957000	3.406024000	-0.403459000

H	4.506518000	3.705442000	-1.898336000
H	5.359028000	5.775151000	-0.831088000
H	4.956559000	6.188774000	1.585885000
H	3.703887000	4.529864000	2.946684000
H	2.856661000	2.448886000	1.901426000
H	-2.856586000	-2.448919000	1.901425000
H	-3.703828000	-4.529900000	2.946664000
H	-4.956577000	-6.188754000	1.585867000
H	-5.359108000	-5.775071000	-0.831086000
H	-4.506584000	-3.705357000	-1.898314000
H	-2.857038000	2.448523000	1.901357000
H	-4.507115000	3.704635000	-1.898457000
H	-5.359985000	5.774240000	-0.831294000
H	-4.957589000	6.188032000	1.585663000
H	-3.704627000	4.529397000	2.946529000

1^{2+}-T

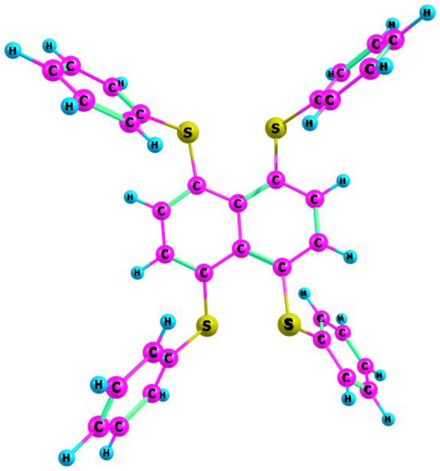

S	-2.976861000	-1.135675000	0.858102000
S	-3.134808000	1.189239000	-0.777014000
S	3.095271000	-1.702307000	-0.981486000
S	3.178742000	1.149888000	-1.397218000
C	0.771839000	-0.147045000	-0.621973000
C	0.736024000	2.269018000	-1.088292000
C	-3.682126000	2.571692000	0.184701000
C	-0.653223000	2.299776000	-0.962586000
C	1.420621000	-1.422171000	-0.455322000
C	1.446863000	1.075913000	-0.975133000

C	-3.895844000	-2.425062000	0.066335000
C	-5.418902000	4.226334000	0.482626000
C	3.765811000	2.629716000	-0.607233000
C	-1.316461000	-1.231241000	0.184782000
C	-1.350831000	1.151433000	-0.627680000
C	4.716635000	3.376798000	-1.328003000
C	-5.816934000	-3.883593000	0.225361000
C	4.934326000	4.868398000	0.566670000
C	3.606578000	-2.625213000	1.614869000
C	-3.613556000	-2.906879000	-1.225104000
C	0.715159000	-2.521706000	0.026482000
C	-4.774592000	4.678598000	1.639543000
C	3.997589000	4.112003000	1.284380000
C	3.416003000	2.985880000	0.709288000
C	-3.586008000	4.071696000	2.070397000
C	-5.003505000	-2.906743000	0.793807000
C	-4.436450000	-3.884990000	-1.774939000
C	5.292719000	4.498543000	-0.734840000
C	-3.035063000	3.014261000	1.352905000
C	4.657891000	-3.793619000	-0.262595000
C	-0.661310000	-0.077587000	-0.358157000
C	-4.881353000	3.170907000	-0.250376000
C	-5.536088000	-4.374477000	-1.054978000
C	-0.631931000	-2.420509000	0.374181000
C	5.315207000	-4.630678000	0.637630000
C	5.119999000	-4.474705000	2.014467000
C	3.792662000	-2.799787000	0.230750000
C	4.266898000	-3.473703000	2.498965000
H	-5.374619000	2.825130000	-1.153884000
H	-6.336383000	4.698676000	0.147475000
H	-5.197455000	5.500677000	2.207907000
H	-3.095805000	4.419229000	2.974124000
H	-2.123089000	2.538514000	1.697319000
H	-1.183419000	3.222476000	-1.172366000
H	1.256618000	3.181746000	-1.354219000
H	-1.137735000	-3.283167000	0.794467000
H	1.216662000	-3.475589000	0.141706000
H	-2.771274000	-2.523748000	-1.790957000
H	-4.227791000	-4.262293000	-2.770850000

H	-6.172691000	-5.135769000	-1.494098000
H	-6.665449000	-4.265481000	0.783374000
H	-5.214028000	-2.531974000	1.791078000
H	2.711666000	2.385560000	1.275629000
H	3.735974000	4.392866000	2.299541000
H	5.389886000	5.739999000	1.025543000
H	6.017263000	5.085609000	-1.289596000
H	4.988448000	3.091681000	-2.339754000
H	2.966838000	-1.834425000	1.992410000
H	4.803051000	-3.916895000	-1.331454000
H	5.975589000	-5.406227000	0.263769000
H	5.637243000	-5.126648000	2.711020000
H	4.131156000	-3.345814000	3.568106000

2

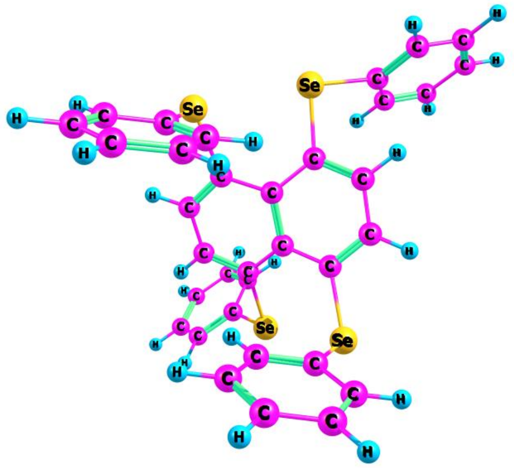

C	2.149621000	2.376778000	-2.488035000
C	2.258320000	2.955318000	-1.218106000
H	2.367230000	2.326807000	-0.340767000
C	2.219139000	4.344340000	-1.081528000
H	2.289052000	4.788291000	-0.092588000
C	2.107828000	5.161221000	-2.211433000
H	2.087433000	6.241655000	-2.101900000
C	2.025117000	4.583824000	-3.480524000
H	1.940243000	5.211987000	-4.362823000
C	2.044128000	3.192263000	-3.619432000
H	1.961564000	2.744042000	-4.604844000
C	0.655919000	-0.039158000	-1.753665000

C	-0.513003000	-0.115025000	-2.476895000
H	-0.491272000	0.028157000	-3.552058000
C	-1.737817000	-0.379481000	-1.840066000
H	-2.620996000	-0.537709000	-2.447871000
C	-1.819088000	-0.442280000	-0.464734000
C	-0.643816000	-0.218566000	0.333196000
C	0.643079000	-0.219156000	-0.332982000
C	-3.404343000	-3.469456000	-1.272037000
H	-2.572018000	-3.728669000	-0.624890000
C	-3.844643000	-4.365807000	-2.247058000
H	-3.345705000	-5.323658000	-2.365455000
C	-4.929775000	-4.034313000	-3.066843000
H	-5.273768000	-4.734542000	-3.822615000
C	-5.576346000	-2.806821000	-2.904076000
H	-6.422325000	-2.547928000	-3.534719000
C	-5.130205000	-1.903512000	-1.932771000
H	-5.626511000	-0.945351000	-1.809769000
C	-4.042461000	-2.232682000	-1.118028000
C	3.403212000	-3.468457000	1.276814000
H	2.570633000	-3.728321000	0.630255000
C	3.843518000	-4.363573000	2.252978000
H	3.344354000	-5.321125000	2.372829000
C	4.928925000	-4.031229000	3.072047000
H	5.272915000	-4.730510000	3.828696000
C	5.575770000	-2.804120000	2.907449000
H	6.421987000	-2.544597000	3.537514000
C	5.129676000	-1.902077000	1.934951000
H	5.626266000	-0.944261000	1.810425000
C	4.041591000	-2.232060000	1.120969000
C	1.818299000	-0.442002000	0.465274000
C	1.737050000	-0.376666000	1.840487000
H	2.620187000	-0.533911000	2.448594000
C	0.512300000	-0.110768000	2.476852000
H	0.490651000	0.034410000	3.551752000
C	-0.656646000	-0.036071000	1.753553000
C	-2.148342000	2.382028000	2.484953000
C	-2.257409000	2.959405000	1.214524000
H	-2.367715000	2.330122000	0.337914000
C	-2.217047000	4.348247000	1.076513000

H	-2.287327000	4.791263000	0.087181000
C	-2.104038000	5.166156000	2.205509000
H	-2.082678000	6.246459000	2.094875000
C	-2.020948000	4.589950000	3.475117000
H	-1.934804000	5.218916000	4.356720000
C	-2.041199000	3.198550000	3.615446000
H	-1.958320000	2.751248000	4.601252000
Se	2.251487000	0.452541000	-2.735709000
Se	-3.508177000	-1.023603000	0.314591000
Se	3.507329000	-1.024785000	-0.313183000
Se	-2.252029000	0.458154000	2.734647000

2^{2+}-CS

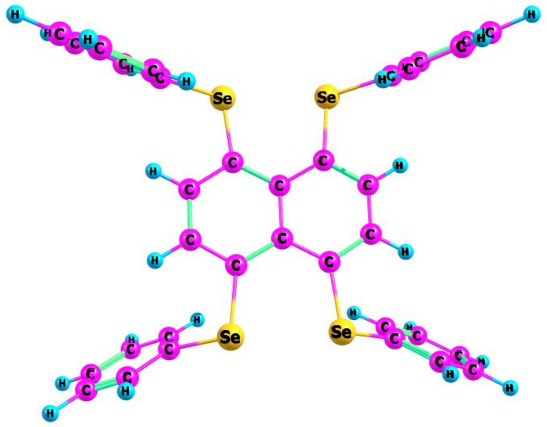

Se	-3.178584000	-1.479494000	-0.885167000
C	-0.669958000	2.466637000	-0.141577000
C	-0.709071000	0.000079000	-0.203376000
C	-1.376471000	1.263651000	-0.335609000
C	-1.376699000	-1.263384000	-0.335549000
C	-3.653671000	3.224109000	1.371523000
C	-4.138310000	-4.174640000	-0.828667000
C	-3.650642000	-3.134914000	-0.027671000
C	-4.137601000	4.175415000	-0.828472000
C	-3.649912000	3.135666000	-0.027519000
C	-4.599531000	5.340152000	-0.211356000
C	-4.113349000	4.394010000	1.973689000

C	-4.586400000	-5.448365000	1.183288000
C	-4.600622000	-5.339240000	-0.211576000
C	-4.584923000	5.449389000	1.183496000
Se	-3.178383000	1.480091000	-0.885005000
Se	3.178261000	1.479534000	0.885484000
C	0.669824000	-2.466589000	0.141278000
C	0.708848000	-0.000034000	0.203298000
C	1.376268000	-1.263595000	0.335501000
C	1.376488000	1.263416000	0.335484000
C	3.653685000	-3.223715000	-1.371349000
C	4.138876000	4.174310000	0.828201000
C	3.650709000	3.134580000	0.027508000
C	4.138019000	-4.175017000	0.828563000
C	3.649984000	-3.135355000	0.027701000
C	4.600218000	-5.339594000	0.211350000
C	4.113630000	-4.393459000	-1.973610000
C	4.586984000	5.447484000	-1.184099000
C	4.601444000	5.338634000	0.210785000
C	4.585543000	-5.448756000	-1.183507000
Se	3.178035000	-1.480005000	0.885363000
C	-3.654804000	-3.223234000	1.371378000
C	-4.114844000	-4.393006000	1.973519000
C	-0.670366000	-2.466486000	-0.141578000
C	0.670228000	2.466524000	0.141301000
C	3.654622000	3.222624000	-1.371562000
C	4.114929000	4.392121000	-1.974027000
H	1.186587000	-3.413100000	0.239734000
H	-1.187239000	-3.412923000	-0.240161000
H	1.187128000	3.412954000	0.239800000
H	-1.186691000	3.413156000	-0.240110000
H	-3.316454000	2.391711000	1.980659000
H	-4.121691000	4.476215000	3.055858000
H	-4.953358000	6.354136000	1.656451000
H	-4.971811000	6.158155000	-0.819625000
H	-4.151169000	4.088333000	-1.910512000
H	-4.123492000	-4.475118000	3.055693000
H	-4.955125000	-6.353005000	1.656222000
H	-4.972886000	-6.157227000	-0.819876000
H	-4.151569000	-4.087648000	-1.910718000

H	-3.317605000	-2.390844000	1.980534000
H	4.151630000	-4.087999000	1.910608000
H	4.972756000	-6.157533000	0.819547000
H	4.954190000	-6.353377000	-1.656539000
H	4.121921000	-4.475602000	-3.055784000
H	3.316205000	-2.391373000	-1.980416000
H	3.317033000	2.390223000	-1.980487000
H	4.123391000	4.474014000	-3.056219000
H	4.955911000	6.351910000	-1.657285000
H	4.974092000	6.156623000	0.818848000
H	4.152314000	4.087537000	1.910267000

2^{2+}-OS

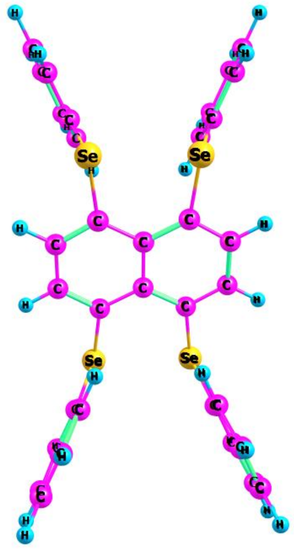

Se	-2.920912000	-1.484563000	-1.580588000
C	-0.629489000	2.455258000	-0.310920000
C	-0.654696000	0.000906000	-0.323285000
C	-1.257778000	1.266592000	-0.613551000
C	-1.257927000	-1.264789000	-0.613223000
C	-3.822957000	1.866660000	1.177187000
C	-5.330031000	-2.589457000	-0.609854000
C	-4.122163000	-2.011181000	-0.187217000
C	-5.330272000	2.589285000	-0.603206000
C	-4.121682000	2.010798000	-0.182903000
C	-6.244218000	3.020164000	0.357112000
C	-4.738593000	2.319274000	2.125336000
C	-5.953405000	-2.892092000	1.711587000

C	-6.245121000	-3.021732000	0.348756000
C	-5.950608000	2.889073000	1.719395000
Se	-2.922119000	1.486605000	-1.578613000
Se	2.922294000	1.486737000	1.578410000
C	0.629003000	-2.453444000	0.312151000
C	0.654666000	0.000916000	0.323651000
C	1.257391000	-1.264780000	0.614627000
C	1.258126000	1.266616000	0.613092000
C	3.823650000	-1.868621000	-1.173370000
C	5.331288000	2.587594000	0.602999000
C	4.122403000	2.009713000	0.182708000
C	5.329911000	-2.588279000	0.609129000
C	4.121478000	-2.010714000	0.187121000
C	6.244572000	-3.020428000	-0.349939000
C	4.740048000	-2.322417000	-2.120224000
C	5.952133000	2.886204000	-1.719625000
C	6.245612000	3.017617000	-0.357342000
C	5.951898000	-2.891359000	-1.712620000
Se	2.920851000	-1.484574000	1.581188000
C	-3.825289000	-1.868526000	1.173424000
C	-4.742102000	-2.322426000	2.119820000
C	-0.630235000	-2.453452000	-0.309305000
C	0.630221000	2.455269000	0.309618000
C	3.823785000	1.865267000	-1.177368000
C	4.739836000	2.316997000	-2.125541000
H	1.099316000	-3.401606000	0.550388000
H	-1.100998000	-3.401624000	-0.546616000
H	1.101153000	3.403452000	0.546548000
H	-1.100151000	3.403426000	-0.548439000
H	-2.885418000	1.428711000	1.500390000
H	-4.509150000	2.222112000	3.181784000
H	-6.661228000	3.237842000	2.461634000
H	-7.179390000	3.470435000	0.040439000
H	-5.553332000	2.715348000	-1.658863000
H	-4.514129000	-2.226363000	3.176687000
H	-6.664951000	-3.241880000	2.452458000
H	-7.179747000	-3.471888000	0.030309000
H	-5.551647000	-2.714341000	-1.665953000
H	-2.888344000	-1.430627000	1.498389000

H	5.552266000	-2.712755000	1.665123000
H	7.179617000	-3.470047000	-0.031966000
H	6.663109000	-3.241065000	-2.453851000
H	4.511312000	-2.226820000	-3.176969000
H	2.886258000	-1.431319000	-1.497862000
H	2.886035000	1.427759000	-1.500553000
H	4.510486000	2.219594000	-3.181987000
H	6.663071000	3.234291000	-2.461881000
H	7.181004000	3.467452000	-0.040695000
H	5.554258000	2.713906000	1.658646000

$\mathbf{2}^{2+}$-T

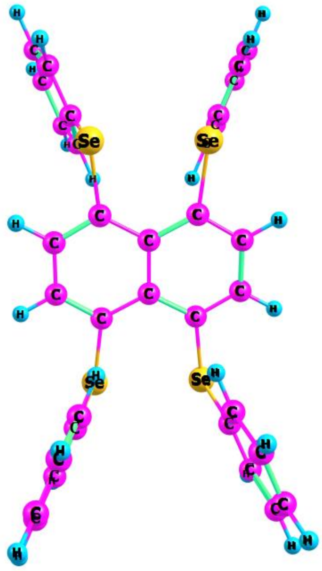

Se	-2.920545000	-1.485382000	-1.581977000
C	-0.629560000	2.454307000	-0.310809000
C	-0.654551000	0.000090000	-0.323249000
C	-1.257443000	1.265854000	-0.614292000
C	-1.257309000	-1.265679000	-0.614548000
C	-3.825409000	1.855940000	1.174299000
C	-5.343651000	-2.554387000	-0.606869000
C	-4.127207000	-1.992841000	-0.186205000
C	-5.343860000	2.554291000	-0.606309000
C	-4.127391000	1.992702000	-0.185774000
C	-6.262094000	2.976071000	0.353919000
C	-4.745837000	2.299174000	2.122280000
C	-5.965316000	-2.852787000	1.715635000

C	-6.261875000	-2.976407000	0.353263000
C	-5.965517000	2.852177000	1.716263000
Se	-2.920715000	1.485576000	-1.581659000
Se	2.920804000	1.485650000	1.581894000
C	0.629329000	-2.454124000	0.311644000
C	0.654562000	0.000094000	0.323590000
C	1.257372000	-1.265671000	0.614811000
C	1.257298000	1.265864000	0.614941000
C	3.825038000	-1.856084000	-1.173982000
C	5.344147000	2.553186000	0.605797000
C	4.127251000	1.992254000	0.185617000
C	5.344205000	-2.553125000	0.606530000
C	4.127369000	-1.992277000	0.186067000
C	6.262432000	-2.974776000	-0.353760000
C	4.745484000	-2.299180000	-2.122016000
C	5.965060000	2.851259000	-1.716951000
C	6.262211000	2.974730000	-0.354693000
C	5.965513000	-2.851464000	-1.716083000
Se	2.920703000	-1.485413000	1.582062000
C	-3.825239000	-1.856354000	1.173899000
C	-4.745658000	-2.299826000	2.121778000
C	-0.629312000	-2.454127000	-0.311279000
C	0.629126000	2.454311000	0.312025000
C	3.824700000	1.855892000	-1.174368000
C	4.744971000	2.298907000	-2.122610000
H	1.099418000	-3.402334000	0.550067000
H	-1.099429000	-3.402339000	-0.549642000
H	1.099163000	3.402523000	0.550543000
H	-1.099785000	3.402517000	-0.548965000
H	-2.881703000	1.431613000	1.497648000
H	-4.513880000	2.207857000	3.178702000
H	-6.679696000	3.193961000	2.458335000
H	-7.203287000	3.413517000	0.037092000
H	-5.569633000	2.674901000	-1.662038000
H	-4.513713000	-2.208723000	3.178221000
H	-6.679487000	-3.194757000	2.457629000
H	-7.203049000	-3.413820000	0.036335000
H	-5.569412000	-2.674781000	-1.662625000
H	-2.881552000	-1.432062000	1.497346000

H	5.570262000	-2.673288000	1.662248000
H	7.203907000	-3.411660000	-0.036993000
H	6.679689000	-3.193174000	-2.458192000
H	4.513253000	-2.208308000	-3.178416000
H	2.881068000	-1.432305000	-1.497270000
H	2.880674000	1.432076000	-1.497449000
H	4.512559000	2.207915000	-3.178960000
H	6.679100000	3.192899000	-2.459222000
H	7.203729000	3.411673000	-0.038138000
H	5.570375000	2.673491000	1.661464000

3^{2+}-cs

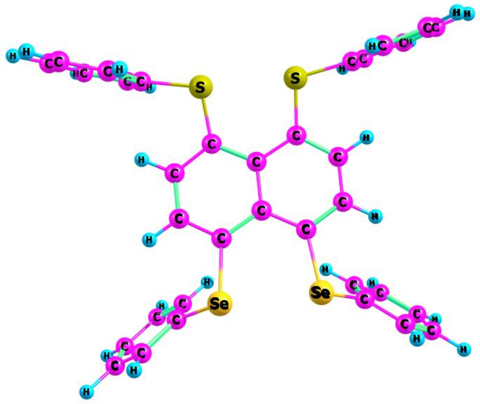

Se	2.770509000	1.463544000	-1.069378000
C	0.256388000	-2.456700000	-0.361297000
C	0.259874000	-0.000149000	-0.587857000
C	0.953509000	-1.253663000	-0.591450000
C	0.953360000	1.253444000	-0.593716000
C	3.072683000	-2.825569000	1.472706000
C	4.073121000	3.929263000	-0.481637000
C	3.314817000	2.893900000	0.084257000
C	4.073483000	-3.929001000	-0.469893000
C	3.314089000	-2.892589000	0.092607000
C	4.565102000	-4.930825000	0.367674000
C	3.562822000	-3.836909000	2.295040000
C	4.313335000	4.890369000	1.729588000
C	4.565870000	4.932936000	0.353051000
C	4.310377000	-4.885425000	1.743719000
Se	2.771238000	-1.464784000	-1.064893000
S	-3.614827000	-1.433992000	-0.201604000

C	-1.106758000	2.467727000	-0.247624000
C	-1.195972000	-0.000048000	-0.380034000
C	-1.869880000	1.276228000	-0.294077000
C	-1.870004000	-1.276321000	-0.294570000
C	-4.077256000	3.885807000	-1.400171000
C	-4.260000000	-3.819575000	1.023234000
C	-3.952199000	-3.198275000	-0.194664000
C	-4.250404000	3.817433000	1.037042000
C	-3.951960000	3.197989000	-0.184156000
C	-4.645947000	5.157300000	1.037464000
C	-4.474425000	5.224166000	-1.381995000
C	-4.756578000	-5.858339000	-0.188118000
C	-4.655629000	-5.159428000	1.018572000
C	-4.756103000	5.858079000	-0.167354000
S	-3.614495000	1.433735000	-0.196985000
C	3.075605000	2.829708000	1.464867000
C	3.566837000	3.842883000	2.284287000
C	0.256316000	2.456780000	-0.364987000
C	-1.106834000	-2.467670000	-0.245565000
C	-4.068259000	-3.884230000	-1.412699000
C	-4.465555000	-5.222578000	-1.399612000
H	-1.617235000	3.416013000	-0.140631000
H	0.796528000	3.395403000	-0.333087000
H	-1.617331000	-3.415869000	-0.137872000
H	0.796711000	-3.395171000	-0.326983000
H	2.518384000	-1.995965000	1.899103000
H	3.382203000	-3.798695000	3.364444000
H	4.702823000	-5.664884000	2.389013000
H	5.145255000	-5.745351000	-0.053612000
H	4.269149000	-3.965363000	-1.537285000
H	3.387925000	3.806880000	3.354055000
H	4.706661000	5.671244000	2.372630000
H	5.145207000	5.746681000	-0.070858000
H	4.267084000	3.963425000	-1.549411000
H	2.522181000	2.000874000	1.893885000
H	-4.181176000	3.264815000	1.968681000
H	-4.878644000	5.646811000	1.977707000
H	-5.074684000	6.895608000	-0.161322000
H	-4.576720000	5.765279000	-2.317219000

H	-3.880879000	3.384119000	-2.342552000
H	-3.864804000	-3.381058000	-2.352789000
H	-4.560781000	-5.762230000	-2.336426000
H	-5.075231000	-6.895862000	-0.186108000
H	-4.895473000	-5.650389000	1.956259000
H	-4.197686000	-3.268443000	1.956236000

3^{2+}-T

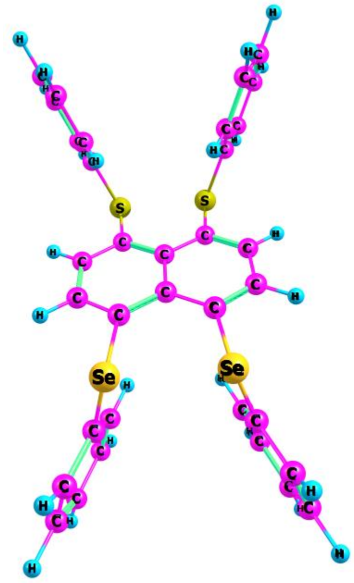

Se	2.512703000	1.484988000	-1.481323000
C	0.263410000	-2.454303000	-0.132935000
C	0.291866000	-0.000021000	-0.142255000
C	0.887678000	-1.265759000	-0.448883000
C	0.887603000	1.265720000	-0.449012000
C	3.542346000	-1.888201000	1.222837000
C	4.929977000	2.670449000	-0.627927000
C	3.765391000	2.048061000	-0.149915000
C	4.930098000	-2.670342000	-0.627566000
C	3.765495000	-2.047913000	-0.149649000
C	5.874469000	-3.131765000	0.288307000
C	4.484742000	-2.373704000	2.127228000
C	5.653466000	2.988303000	1.662656000
C	5.874348000	3.132012000	0.287875000
C	5.653569000	-2.987880000	1.663066000
Se	2.512809000	-1.485039000	-1.481144000
S	-3.172642000	-1.430423000	1.701369000

C	-0.986208000	2.455091000	0.504695000
C	-1.008817000	-0.000025000	0.523101000
C	-1.615067000	1.266736000	0.820605000
C	-1.615039000	-1.266791000	0.820639000
C	-4.107553000	2.046536000	-0.883250000
C	-5.463339000	-2.681912000	1.054826000
C	-4.299459000	-2.101677000	0.509177000
C	-5.463306000	2.681915000	1.055413000
C	-4.299545000	2.101627000	0.509564000
C	-6.427874000	3.207898000	0.201146000
C	-5.083575000	2.579432000	-1.721140000
C	-6.240844000	-3.159624000	-1.186559000
C	-6.427770000	-3.207882000	0.200397000
C	-6.241205000	3.159591000	-1.185843000
S	-3.172544000	1.430330000	1.701557000
C	3.542260000	1.888523000	1.222594000
C	4.484657000	2.374164000	2.126911000
C	0.263272000	2.454260000	-0.133168000
C	-0.986106000	-2.455141000	0.504861000
C	-4.107205000	-2.046639000	-0.883604000
C	-5.083093000	-2.579524000	-1.721658000
H	-1.456036000	3.402042000	0.748485000
H	0.728285000	3.403103000	-0.378857000
H	-1.455914000	-3.402095000	0.748681000
H	0.728483000	-3.403145000	-0.378518000
H	2.638182000	-1.415424000	1.589836000
H	4.311641000	-2.266007000	3.193288000
H	6.387270000	-3.361184000	2.370102000
H	6.776095000	-3.615967000	-0.072479000
H	5.095705000	-2.806414000	-1.692465000
H	4.311570000	2.266604000	3.192987000
H	6.387167000	3.361715000	2.369634000
H	6.775961000	3.616184000	-0.072985000
H	5.095569000	2.806384000	-1.692846000
H	2.638110000	1.415776000	1.589665000
H	-5.601654000	2.731428000	2.131570000
H	-7.320300000	3.663787000	0.617112000
H	-6.994049000	3.574283000	-1.848281000
H	-4.942296000	2.544503000	-2.796703000

H	-3.216471000	1.596497000	-1.305847000
H	-3.216023000	-1.596657000	-1.306051000
H	-4.941609000	-2.544640000	-2.797196000
H	-6.993579000	-3.574310000	-1.849124000
H	-7.320290000	-3.663727000	0.616212000
H	-5.601888000	-2.731391000	2.130959000

Supplementary References

1. a) Becke A. D., Density–functional thermochemistry. III. The role of exact exchange. *J. Chem. Phys.*, **98**, 5648–5652 (1993); b) Lee C. T., Yang W. T. & Parr R. G., Development of the colle-salvetti correlation-energy formula into a functional of the electron density. *Phys. Rev. B: Condens. Matter Mater. Phys* . **37**, 785–789 (1988).
2. a) Feller D., *J. Comput. Chem.,* The role of databases in support of computational chemistry calculations. 17, 1571-1586 (1996); b) Schuchardt K. L. et al. Basis set exchange: a community database for computational sciences. *J. Chem. Inf. Model.*, **47**, 1045-1052 (2007).
3. Frisch, M. J.; Trucks, G. W.; Schlegel, H. B.; Scuseria, G. E.; Robb, M. A.; Cheeseman, J. R.; Scalmani, G.; Barone, V.; Mennucci, B.; Petersson, G. A.; Nakatsuji, H.; Caricato, M.; Li, X.; Hratchian, H. P.; Izmaylov, A. F.; Bloino, J.; Zheng, G.; Sonnenberg, J. L.; Hada, M.; Ehara, M.; Toyota, K.; Fukuda, R.; Hasegawa, J.; Ishida, M.; Nakajima, T.; Honda, Y.; Kitao, O.; Nakai, H.; Vreven, T.; Montgomery, Jr., J. A.; Peralta, J. E.; Ogliaro, F.; Bearpark, M.; Heyd, J. J.; Brothers, E.; Kudin, K. N.; Staroverov, V. N.; Kieth, T.; Kobayashi, R.; Normand, J.; Raghavachari, K.; Rendell, A.; Burant, J. C.; Iyengar, S. S.; Tomasi, J.; Cossi, M.; Rega, N.; Millam, N. J.; Klene, M.; Knox, J. E.; Cross, J. B.; Bakken, V.; Adamo, C.; Jaramillo, J.; Gomperts, R.; Stratmann, R. E.; Yazyev, O.; Austin, A. J.; Cammi, R.; Pomelli, C.; Ochterski, J. W.; Martin, R. L.; Morokuma, K.; Zakrzewski, V. G.; Voth, G. A.; Salvador, P.; Dannenberg, J. J.; Dapprich, S.; Daniels, A. D.; Farkas, Ö.; Foresman, J. B.; Ortiz, J. V.; Cioslowski, J.; and Fox, D. J., Gaussian 16, Revision A.03. Gaussian, Inc.: Wallingford CT, 2016.
4. Bader R. F.W., *Atoms in molecules. A quantum theory,* Oxford University Press, Oxford, 1990.
5. Hegarty, D. & Robb, M. A. Application of unitary group-methods to configuration-interaction calculations. *Mol. Phys.*, **38**, 795-812 (1979).
6. Yamamoto, N., Vreven, T., Robb, M. A., Frisch, M. J., & Schlegel, H. B. A Direct Derivative MC-SCF Procedure. *Chem. Phys. Lett.*, **250**, 373-378 (1996).
7. Neese, F. The ORCA program system. WIREs Comput. *Mol. Sci.* **2**, 73–78 (2012).
